# Autogenous Healing of Early-Age Cementitious Materials Incorporating Superabsorbent Polymers Exposed to Wet/Dry Cycles

**DOI:** 10.3390/ma11122476

**Published:** 2018-12-06

**Authors:** Yewon Shim, Geuntae Hong, Seongcheol Choi

**Affiliations:** Department of Civil and Environmental Engineering, Chung-Ang University, 84 Heukseok-ro, Dongjak-gu, Seoul 06974, Korea; yewon0819@hanmail.net (Y.S.); hgt0916@naver.com (G.H.)

**Keywords:** autogenous healing, capillary water absorption testing, cementitious materials, superabsorbent polymer, wet/dry cycling

## Abstract

This study experimentally investigated the autogenous healing performances of cementitious materials incorporating superabsorbent polymers (SAPs) after exposure to eight cycles of wet/dry conditions. In each cycle, cracked cement paste specimens with different SAP dosages were exposed to wet conditions for 1 h, during which capillary water absorption tests were conducted, and then exposed to dry conditions for 47 h. The test results reveal that the initial sorptivity values of the reference, 0.5% SAP, 1.0% SAP, and 1.5% SAP specimens after one cycle were decreased by 22.9%, 36.8%, 42.8%, and 46.3%, respectively, after eight cycles. X-ray micro-computed tomography analysis showed that the crack volume percentages filled with healing products were 1.1%, 1.6%, 2.2%, and 2.9% in the reference, 0.5% SAP, 1.0% SAP, and 1.5% SAP specimens, respectively. As the cycling was repeated, the reduction ratio of the initial sorptivity and the quantity of healing products were increased with increases in SAP dosage. Furthermore, more healing products were distributed near SAP voids than in other sections in the specimens. This study demonstrates that the incorporation of SAPs in cementitious materials can enhance the autogenous healing performances of materials exposed to cyclic wet/dry conditions.

## 1. Introduction

Superabsorbent polymers (SAPs) are hydrogel materials capable of absorbing and retaining moisture by osmotic pressure that can swell to several hundred times their own dry weight. This water absorption capacity enables the use of SAPs as internal curing agents to mitigate the autogenous and drying shrinkage of concrete, which is primarily related to moisture loss [1]. Existing studies [2,3,4,5] suggest that SAPs are also effective in enhancing the self-healing performances of cementitious materials by supplying water to cracked cement matrices when a material is exposed to various environments, including dryness. Most concrete structures in the field are exposed to the atmosphere, where the temperature and relative humidity (RH) vary. The restrained volume changes due to variations in temperature and RH generally accompany early-age cracks in cast-in-place concrete structures [6]. Even though several studies have analyzed the improvement in the autogenous healing of cracks occurring within 28 days by SAPs [3,7,8,9], there are relatively fewer evaluations on the autogenous healing performances of SAP-incorporating cementitious materials during hardening stages. The autogenous healing of hardening cementitious materials, which is the outcome of further hydration of unhydrated cement particles [9,10], can be promoted by the internal curing effects of SAPs, when the materials are exposed to the atmosphere. Cracks lead to deterioration of durability and corresponding reduction in the service life of concrete structures [6]. On this basis, considering the importance of controlling cracks at an early age, it is necessary to evaluate the internal curing effect of SAPs on the autogenous healing of early-age cementitious materials, when exposed to wet/dry cycling in an approximation of field conditions. 

Among the experimental methods that evaluate the self-healing performance of cementitious materials, the water flow test, which estimates the change in water permeability through cracks in cementitious materials, is the most commonly used [11]. However, because the permeability of cementitious materials with cracks of <50 μm in width is similar to that of sound materials [12], the water permeability test may show limited accuracy for specimens containing very narrow cracks [13]. In addition, the self-healing products formed on the crack surface can be lost in water runoff through the cracks, especially under repeated testing [14]. The capillary water absorption test, which is generally used in evaluating the durability of concrete [15], can be also used to evaluate the self-healing performances of cementitious materials by measuring the water absorption rate, i.e., sorptivity [16,17]. Because cracks provide preferential flow paths with easy access to the cement matrix, the sorptivity is higher in specimens with cracks than in those without cracks. The preferential flow path of water is blocked when cracks are healed, and the sorptivity by capillary phenomena is reduced accordingly [9]. In addition, water absorption by capillary phenomena continues with fine cracks and flexural cracks of <50 μm in width and the loss of healing products can be avoided even with repeated testing. 

Consequently, the purpose of this study is the experimental and quantitative evaluation of the autogenous healing performances of early-age SAP-containing cementitious materials exposed to wet/dry cyclic conditions. Cement paste specimens incorporating varying dosages of SAPs were prepared and intentionally cracked in the flexural mode at the age of 7 days, yielding cracks typical to concrete structures. In the cyclic testing, each cycle consisted of 1 h wetting and 47 h drying at the constant temperature and relative humidity (RH) of 25 °C and 50%, respectively, throughout the test. Capillary water absorption testing was performed in the wetting stage of each cycle. The test was repeated over eight cycles. The crack autogenous healing performance was evaluated as the reduction ratio of the initial sorptivity over wet/dry cycling. In addition, X-ray micro-computed tomography (CT) was performed, quantifying the healing products by analyzing the changes in crack volume before the first cycle and after eight cycles. The autogenous healing performance was also evaluated by estimating the healing ratio, expressed as a percentage of the volume of healing products to the volume of the original crack before healing. The spatial distributions of healing products in the specimens after healing were compared with those of SAP voids; the correlation between the ratio of initial sorptivity reduction and the amount of healing product formed in the specimens was analyzed.

## 2. Materials and Methods

### 2.1. Materials

In the test, cement paste specimens were prepared using Type I Portland cement and SAPs. The cement satisfied ASTM C150 requirements, and its fineness and density were 3499 cm^2^/g and 3.31 g/cm^3^, respectively. Table 1 shows the chemical composition of the cement used in the study.

**Figure 1:**
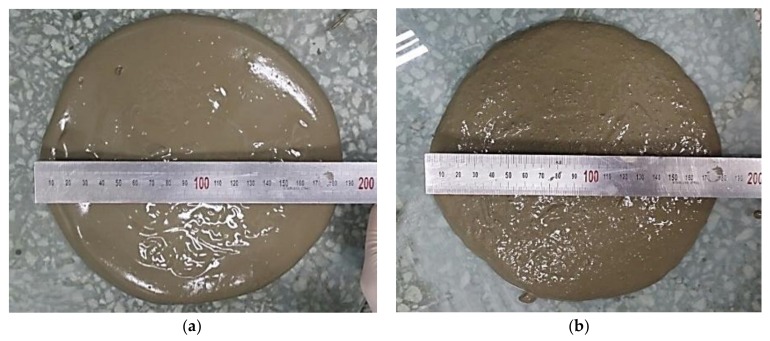
Flow measurement of (**a**) reference specimen (REF) and (**b**) superabsorbent polymers (SAPs)-added mixture.

Irregularly shaped SAP (poly acrylic acid sodium salt), density: 1.66 g/cm^3^, LG Chem, Seoul, Korea) was used in the study. Table 2 shows the mixture proportions of the cement paste specimens for the capillary water absorption test and X-ray micro-CT analysis. The water-to-cement ratio is 0.35 and the SAP dosages, which are the experimental variables, are 0.5%, 1.0%, and 1.5% of the weight of cement. The number following “S-” in the specimen name refers to the SAP dosage (%). To the cement mixtures, 10 g of extra water per 1 g SAP was added, having been determined as the amount of extra water necessary to allow all specimens with SAPs to exhibit the same flow characteristics as the reference specimen (REF) without SAPs [18,19,20,21,22]. Flow tests were conducted in accordance with international standard ASTM C 1437. Figure 1 shows the flow measurement for the REF and SAP-added mixtures.

The materials were mixed in accordance with ASTM C 305, with the addition of dry mixing for 1 min in order to disperse the SAPs and cement uniformly [23]. After mixing, the cement paste mixtures were cast into prism molds (40 × 40 × 160 mm) for the capillary water absorption tests, and cylindrical molds (11 mm in diameter and 10 mm in height) for X-ray micro-CT scanning. The molded mixtures were cured for 7 days in a chamber at the constant temperature of 20 °C and 100% RH. 

### 2.2. Preparation of Specimens

**Figure 2:**
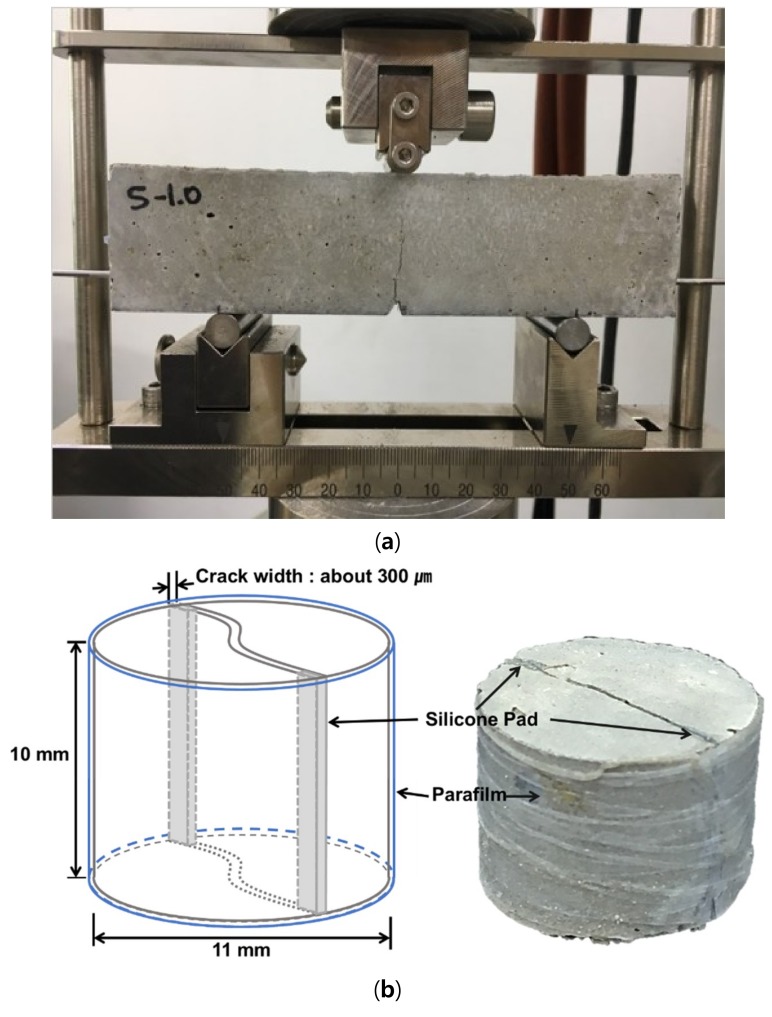
(**a**) Flexural crack-containing specimen for capillary water absorption test; (**b**) cracked specimen for X-ray micro-computed tomography (CT) analysis.

For the capillary water absorption test, a flexural crack was induced in each specimen by three-point-bending using MTS 810 [24]. In order to induce a single flexural crack at the middle of the specimen, a notch is introduced at the center of the bottom of the specimen, as shown in Figure 2a. Two stainless steel bars of 3 mm in diameter were embedded in the specimen at the height of 10 mm in the prism specimens to control the crack width. The maximum crack width of 0.25–0.35 mm occurred at the bottom, and the crack width gradually decreased with increasing height before stopping at the height of approximately 39 mm. Before conducting the capillary water absorption test, the cracked specimens were dried at 25 °C and 50% RH for 3 days. 

Previous studies [21,23] indicated that the amount of healing products was very small compared to the specimen size for the capillary water absorption test. In addition, the accuracy of X-ray CT scanning (Model: Skyscan 1172, Bruker, Konitch, Belgium) is improved with decreased voxel size, which is inversely proportional to the specimen size. Therefore, smaller cylindrical specimens for X-ray micro-CT analysis were separately fabricated, as shown in Figure 2b. After 7 days of standard curing, a through-crack was induced at the center of the specimens by tensile splitting using MTS 810. To prevent the crack width in the specimens from changing under the expansion and contraction of the cement matrix exposed to wet/dry cycles, silicone pads are inserted at both ends of the crack and the specimen is wrapped with Parafilm, as shown in Figure 2b. The average maximum crack width of the specimens is approximately 300 μm, similar to that of the capillary water absorption test specimens. Furthermore, twelve cubic specimens (each 50 × 50 × 50 mm) were prepared for each mixture, and their compressive strengths were measured after 3, 7, and 28 days, in accordance with international standard ASTM C 109.

### 2.3. Capillary Water Absorption Test

The amount of water absorbed by capillary action for a porous material such as a cementitious material in contact with water can be expressed as Equation (1):

(1) $$I=\frac{\Delta W}{A\cdot d}=S\cdot t^{1/2}+I_{0}$$

where *I* denotes the water absorption (mm), Δ*W* denotes the mass of absorbed water (g), *A* denotes the area of the specimen exposed to water (mm^2^), *d* denotes the density of water (g/mm^3^), *t* denotes the measurement time (h), *I*_0_ denotes the correction constant from surface effects at the moment of contact with water [25], and *S* denotes the absorption rate, i.e., sorptivity (mm/h^1/2^), corresponding to the slope of the *I*–*t*^1/2^ graph, which has a bilinear form in general for the initial and secondary sorptivities [26].

**Figure 3:**
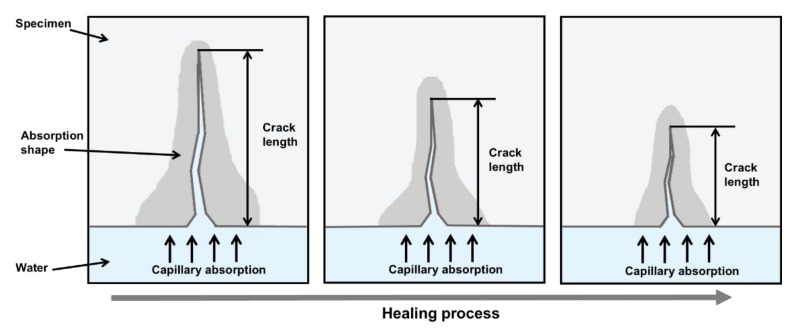
Schematic of change in capillary water absorption through crack as healing progresses.

The initial sorptivity of cementitious materials is significantly affected by capillary pores, crack widths, crack lengths, and tortuosity [27], whereas the secondary sorptivity is generally influenced by the saturation and diffusion of air voids and gel pores that have little relation to cracks [26,28]. Figure 3 illustrates the change in capillary water absorption through cracks as crack healing progresses in a cementitious material. Cracks provide preferential flow paths with easy access to internal capillary pores, thus increasing the initial sorptivity compared to non-cracked specimens [26]. Conversely, because the preferential water flow path is blocked by the healing products, and because the crack is shortened with healing as shown in Figure 3, water absorption by capillary action and the overall sorptivity are reduced [16]. Therefore, changes in sorptivity are directly related to the autogenous healing performances of cracks in cementitious materials [29].

**Figure 4:**
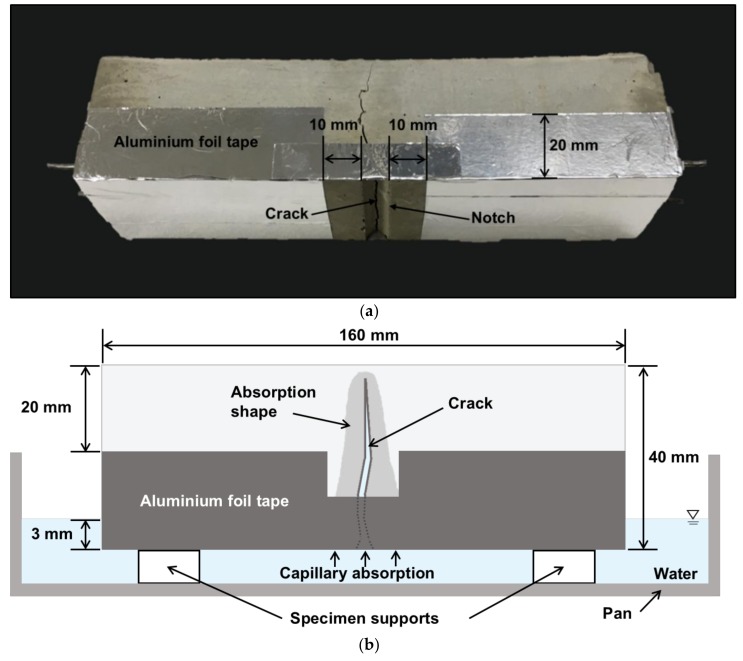
(**a**) Specimen sealed with aluminum foil tape; (**b**) Illustration of capillary water absorption test.

Figure 4a,b show the capillary water absorption test specimen and experimental setup, respectively. The capillary water absorption test conducted in this study used modified total experimental times and measuring intervals from the ASTM C 1585 specification. In ASTM C 1585, the mass change is measured at 1, 5, 10, 20, 30, and 60 m, and then every hour until 6 h have elapsed; the initial sorptivity is evaluated using the slope of mass change for 6 h from the start of the test. 

As this study focuses on analyzing the autogenous healing performance of cementitious materials by observing the water absorption, only the initial sorptivity, which is closely related to crack healing as described above, was evaluated in the test. Furthermore, the slope of the *I*–*t*^1/2^ graph for cementitious materials is generally linear for the first 6 h of exposure to moisture [15]. Therefore, in the capillary water absorption test, the mass change of the specimen was measured for only 1 h with an accuracy of 0.01 g at intervals of 1 min for the first 10 min and of 5 min until 60 min had elapsed. The initial sorptivity was determined using the water absorption from 1 min to 1 h.

As shown in Figure 4a,b, all the surfaces exposed to water are completely sealed with aluminum foil tapes except for 10 mm on both sides of the notch, thus ensuring uniaxial water uptake through the crack. During the capillary water absorption test, the specimen was immersed in distilled water at a depth of 3 ± 1 mm. The water absorption was evaluated by measuring the mass change of the specimens and normalized by the surface area and the density of water, as shown in Equation (1). Thereafter, the specimen was dried in a controlled environmental chamber at a 25 °C and 50% RH for 47 h. These wet/dry cyclic conditions were repeated for eight cycles over 16 days.

### 2.4. X-ray Micro-CT Scanning

X-ray micro-CT scanning was performed before cycling and after eight wet/dry cycles to quantify the amounts of autogenous healing products formed near the cracks in the cylindrical specimens shown in Figure 2b. The cylindrical specimen was rotated with an angle step of 0.3° and images were taken at the output energy of 100 kV and current of 100 μA using an X-ray cone beam. The voxel of the raw images was adjusted to 12.45 μm. All images taken before cycling and after eight cycles were imported into Avizo (Visualization Sciences Group, Bordeaux, France). After filtering using a non-local mean filter to remove white noise under constant conditions, all images were set to regions of interest (ROI) using the same threshold value for each specimen. The healing products were obtained from the crack ROI related to water movement through capillary absorption. 

## 3. Results and Discussion

### 3.1. Capillary Water Absorption Test

**Figure 5:**
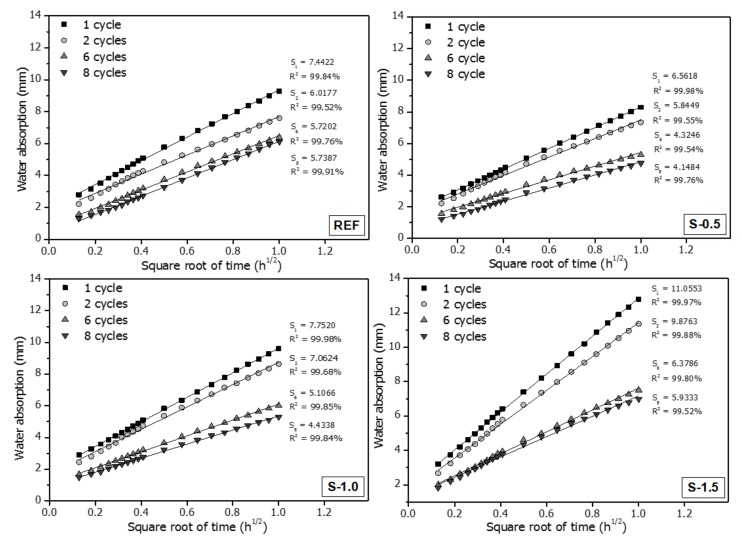
Water absorption of specimens over the square root of time, for different numbers of cycles.

Figure 5 shows the changes in water absorption of the specimens over the square root of time for varying cycles. As soon as the specimens make contact with water, vertical water absorption occurs rapidly through the crack; as time progresses, water absorption spreads horizontally. As shown in Figure 5, the water absorption in all specimens at all cycles is linearly proportional to the square root of time within 1 h. Figure 5 also shows the initial sorptivity at n cycles (*S_n_*), which is the slope of the graph between water absorption and the square root of time, and is calculated using Equation (1). The initial sorptivity decreased as the number of cycles increased. The effect of SAPs on autogenous crack healing in the specimens was observed when the change in the crack was evaluated, in terms of the variations in the initial sorptivity rather than water absorption. This is because the changes in the crack are closely related to the initial sorptivity. The values for the initial sorptivity and water absorption of the S-1.5 specimen at 1 cycle were relatively large compared with those of the REF, S-0.5, and S-1.0 specimens, as shown in Figure 5. Capillary flow through cracks is influenced by crack geometry, such as crack width and orientation, porosity, and tortuosity [30]. It was considered that the internal crack geometry of the S-1.5 specimen was significantly different to those of the REF, S-0.5, and S-1.0 specimens, although similar crack widths and lengths have been induced in all specimens.

The initial sorptivity of cementitious materials is affected by factors including the width, tortuosity, and length of cracks [20], and these factors were not controllable in the test specimens. It was therefore logical to evaluate self-healing performance by comparing the reduction ratio of the initial sorptivity of the specimen at each cycle, rather than comparing the absolute values of the progressive sorptivity measurements.

The reduction ratio of the initial sorptivity is obtained by using Equation (2):

(2) $$R_{n}=\frac{\left(S_{1}-S_{n}\right)}{S_{1}}\times 100(\%)$$

where *R_n_* denotes the reduction ratio of the initial sorptivity at *n* cycles, while *S*_1_ and *S_n_* denote the initial sorptivities measured at one cycle and *n* cycles, respectively, of capillary water absorption testing.

**Figure 6:**
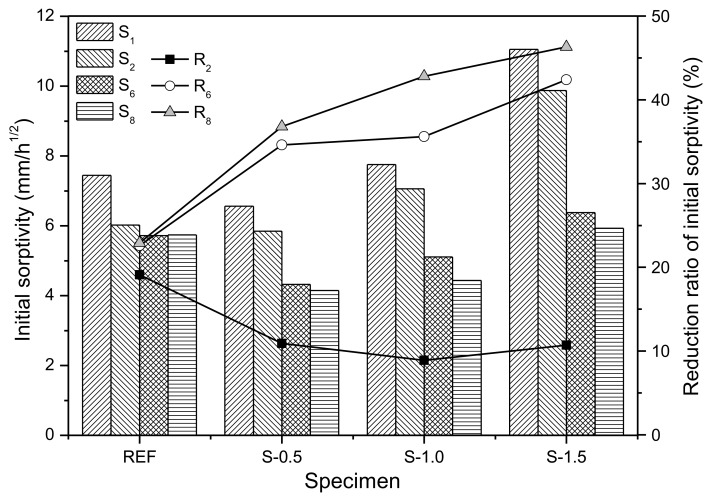
Initial sorptivity and reduction ratios of specimens exposed to wet/dry cycling.

Figure 6 shows the initial sorptivity *S_n_*, which is estimated from the slope of the *I–t^1/2^* graph in Figure 5, and the reduction ratio of the initial sorptivity *R_n_*, for different cycle numbers. Scales for *S_n_* and *R_n_* are shown on the primary and secondary axes, respectively. As shown, *S*_2_ is smaller than *S*_1_ for all specimens. This is because the autogenous healing effect, driven by further hydration of the un-hydrated cement particles in the crack surface, reduces the sorptivity in the second cycle, despite the relatively short period of 48 h between the first and second cycles permitted for crack healing [31,32,33,34]. Because the cracks in the specimens were induced at the early age of 7 days, relatively large amounts of un-hydrated cement particles are present between the first and second cycles during the test; these are actively hydrated once in contact with water. The reduction ratio of initial sorptivity of the reference specimen after two cycles, *R*_2_, is 19.8% whereas those of S-0.5, S-1.0, and S-1.5 specimens are 11.9%, 9.6%, and 11.1%, respectively. This is because relatively less un-hydrated cement is present in the S-series specimens than in REF because of the internal curing effect of SAPs, thus yielding less reduction of *R*_2_ in the S-series specimens than in REF specimen [33]. As the wet/dry cycles are repeated, the reduction ratio of initial sorptivity of REF specimen is slightly increased up to six cycles and relatively unchanged for the sixth to eighth cycles; *R*_8_ is 22.9% for the REF specimen. This is because most of the crack autogenous healing due to additional hydration is developed within 14 days, producing the decrease in the initial sorptivity for the first six cycles, and thereafter the decrease is slight due to negligible autogenous healing of cracks in the REF specimen under wet/dry cycling [27]. On the other hand, *R_n_* of the S-series specimens continuously increases with repeated wet/dry cycling. At the completion of the capillary water absorption test, *R*_8_ is 36.8%, 42.9%, and 46.8% for the S-0.5, S-1.0, and S-1.5 specimens, respectively, suggesting that *R*_8_ increases with increasing SAP dosage. This result reveals that the SAPs located at the crack surface supply the additional moisture necessary for further hydration under wet/dry cycling. More healing products are formed near the crack surfaces of S-series specimens by the internal curing effect of the SAPs, thus decreasing the water absorption into cracks by capillary action [16]. 

**Figure 7:**
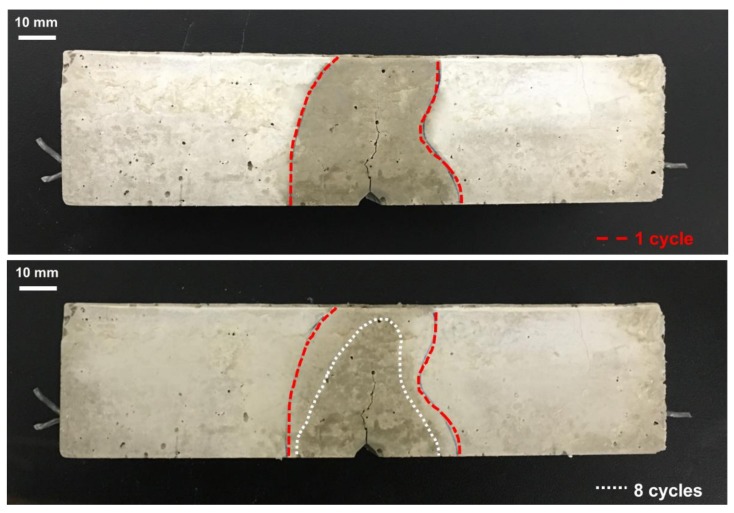
Region of water absorption in the S-0.5 specimen at first and eighth cycles during the capillary water absorption test.

Figure 7 shows the region of water absorption in the S-0.5 specimen after one and eight cycles during the capillary water absorption test. As the wet/dry cycling is repeated, not only the initial sorptivity but also the height and width of the water absorption area are decreased, as shown in Figure 7, which is consistent with the schematic shown in Figure 3. As crack autogenous healing progresses, the crack length is shortened by the filling of the narrow-width crack tip [16], which blocks the vertical moisture penetration path of water, thus decreasing the initial sorptivities of the specimens. In addition, the healing products formed on the crack surface and SAP void surface fill the capillary pores, thus reducing horizontal penetration by the densification of the crack surface [34] and accordingly further decreasing the initial sorptivity. 

### 3.2. X-ray Micro-CT Image Analysis

**Figure 8:**
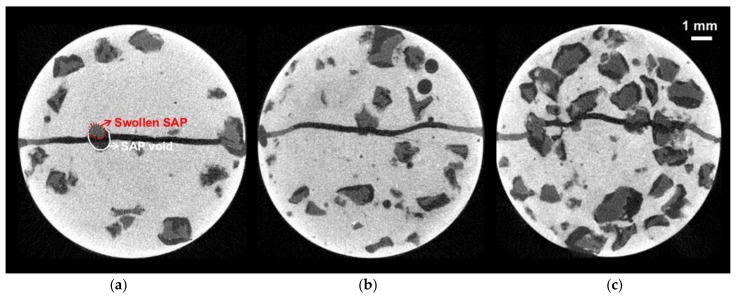
Gray-level X-ray micro CT image of (**a**) S-0.5, (**b**) S-1.0 and (**c**) S-1.5 specimen, taken after cracking.

Figure 8 shows gray level X-ray micro CT images of specimens incorporating SAPs, after cracking. The bright part represents the cement matrix, while blackish parts indicate voids and cracks. The original CT images are composed of 256 gray levels, in which a solid material, such as the cement matrix, appears as a bright color close to 255, while voids and cracks appear as dark colors, approaching 0 [31]. SAPs hold the additional water until after setting, and afterward release the absorbed water for further hydration of mixture [19]. Releasing water from the SAP to the surrounding cement matrix is governed by the RH gradient: when the RH drops, because of hydration [35], then water in the SAPs, which had served as additional water in the mixture, can be released slowly to the surrounding cement matrix, where it can promote further cement hydration. From Power’s model and existing work [36], there was still free capillary water in the matrix for 1–2 days, however after 3 days, the amount of free capillary water in the REF matrix has decreased, and it then became the limiting factor for further hydration. In contrast, SAPs in the S-series specimen matrices continued to release water, promoting further hydration [36].

Figure 8c shows that as more SAP particles were incorporated into the S-1.5 specimens than in the S-0.5 and S-1.0 specimens, more voids, formed by the swelling of SAPs in cement pore solution, were connected to each other. The water absorption and initial sorptivity of S-1.5 in Figure 5 and Figure 6 are relatively high compared to the other specimens, as both the SAPs in cracks, and some of the SAPs inside the cement matrix which was connected to the SAP voids in the cracks, could absorb capillary water. However, as the connection between SAP voids in crack surfaces and SAP voids in the cement matrix gradually became blocked, due to the progress of hydration reactions and formation of healing products [37,38], the initial sorptivity of the S-1.5 specimen noticeably decreased, as the number of wet/dry cycles increased, as shown in Figure 5 and Figure 6.

**Figure 9:**
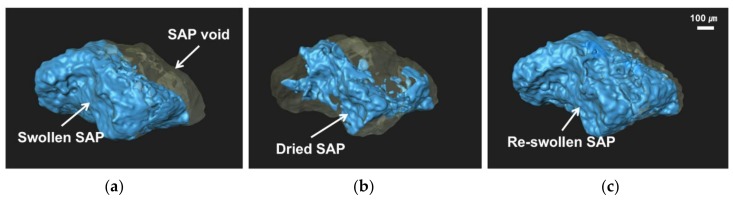
Three-dimensional images of SAP particles and voids in a crack surface of an S-0.5 specimen: (**a**) after cracking; (**b**) after a drying cycle; (**c**) after a wetting cycle.

Figure 9 shows three-dimensional, reconstructed images for the SAP void and SAP particles on the crack surface, shown in the circle of Figure 8a. It shows the swelling behavior of the SAP particle in the void before and after cracking. As shown in Figure 9a, SAP retains some water right after cracking, for at least 7 days, which indicates that only a portion of the additional water absorbed by SAP during mixing was used for the further hydration of the surrounding cement matrix. It can therefore be judged that additional water had only a small effect on the total w/c ratio during a 7-day curing period. After that, as the wet/dry cycle was repeated, the SAP repeatedly deswelled, by releasing water under drying conditions, and swelled, by absorbing ingress water under wetting conditions, as shown in Figure 9b,c. Therefore, it is considered that the long-term, autogenous, crack healing performance by SAP-added, cementitious materials, exposed to wet/dry cyclic conditions, can be improved by the internal curing effect, due to the repeated swelling/deswelling of SAP particles, as shown in Figure 5 and Figure 6.

As shown in Figure 8 and Figure 9, a small amount of absorbed water was released to the surrounding cement matrix, resulting in a reduced SAP volume during curing. Existing studies [37,39,40] showed that, at 7 days, the degree of hydration of SAP-added mixtures, with additional water during curing, was relatively higher than that of REF mixtures without SAP. Therefore, it can be judged that unhydrated cement particles present in the cement matrix around the SAP voids have been further hydrated by the water released from the SAP. In other words, it is considered that the degree of hydration immediately after cracking was higher in SAP-added specimens than in REF specimens, and that the amount of unhydrated cement particles was relatively small in SAP-added specimens, compared to the REF specimens

As a result, it is expected that the degree of hydration of SAP-added specimens immediately after cracking, would be slightly higher than that of the REF specimen. However, since each mixture’s degree of hydration was not quantitatively evaluated at 7 days during curing, further study is needed, in order to quantify the effect of additional water absorbed by SAPs on the degree of hydration, according to the curing age, of the surrounding cement matrix. Applicable methods for this work would include thermal analysis and/or Rietveld quantitative phase analysis [41].

**Figure 10:**
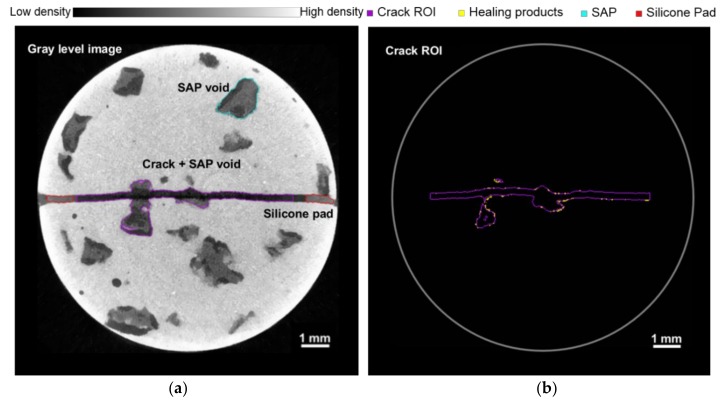
(**a**) Gray-level X-ray micro CT image of specimen. Crack regions of interest (ROI) includes the crack and directly connected SAP voids is shown in purple, the silicone pad in red, and SAP voids in blue; (**b**) The crack ROI (purple) and healing products (yellow).

Figure 10a shows a gray-level X-ray micro-CT image of the specimen obtained before the first cycle. The red regions represent the silicone pads installed at both ends of the crack to control the crack width, as shown in Figure 2b, and the irregularly shaped blue region represents a SAP void in the cement matrix, caused by the swelling and de-swelling of SAPs by absorbing and releasing the mixing water. The purple region represents the crack ROI, including the crack and SAP voids directly connected to it, where the formation of healing products is analyzed in this study. In the crack ROI setting, the regions of the silicone pad are excluded. Figure 10b shows the crack ROI shown in purple and the healing products shown in yellow generated in the crack ROI after eight wet/dry cycles. The healing products are obtained from the segmentation process described below.

**Figure 11:**
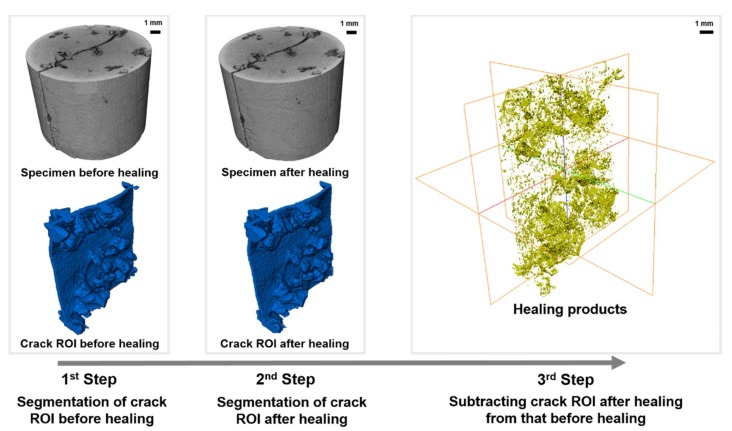
Segmentation of healing products in the crack ROI.

Figure 11 shows the segmentation of healing products from the original cement matrix using images obtained before and after healing. As the healing product volume is much smaller than the specimen volume, imaging and reconstruction conditions such as pixel size, exposure time, and filter are identical before and after healing to reduce errors. The images obtained before and after healing are perfectly aligned using Avizo. After setting the crack ROI for each of the before and after healing images, as shown in Figure 11, two three-dimensional crack ROIs are subtracted from each slice using an arithmetic function to identify the newly generated healing products.

The autogenous healing performance is evaluated in terms of the crack healing ratio, expressed as the percentage of the volume of healing products relative to the crack volume before healing, as shown in Equation (3):

(3) $$H_{r}=\frac{V_{hp}}{V_{c}}\times 100(\%)$$

where *H_r_* denotes the crack healing ratio, *V_c_* denotes the volume of the crack ROI before the first cycle (for S-series specimens, the crack ROI includes directly connected SAP voids), and *V_hp_* denotes the total volume of healing products quantified after eight cycles.

**Figure 12:**
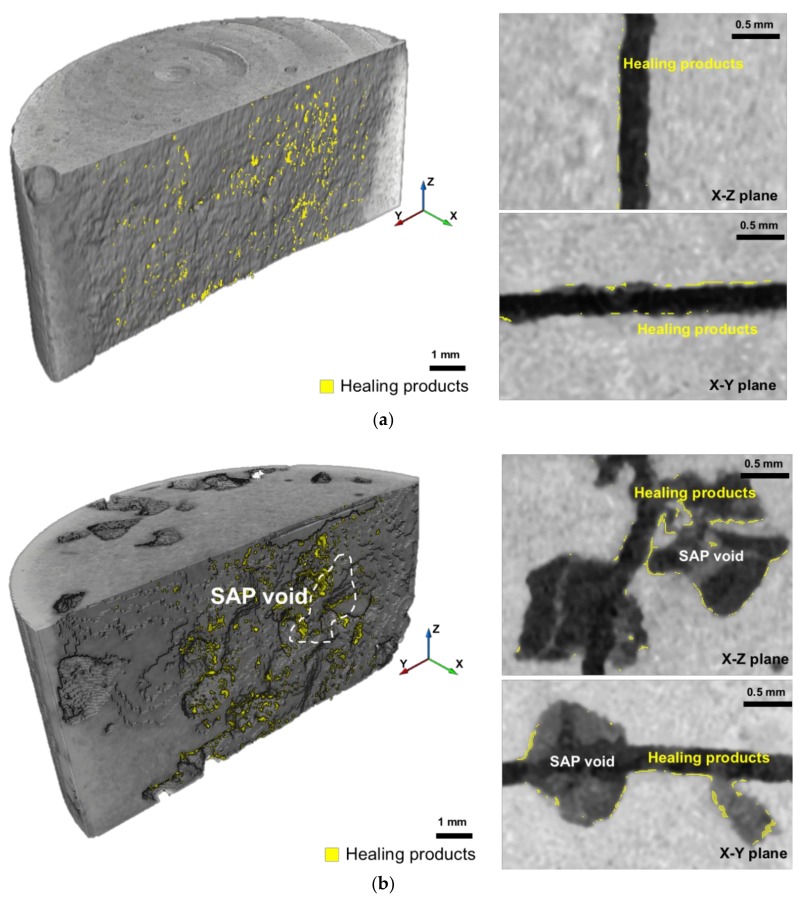
Three-dimensional images of healing products in the (**a**) reference specimen (REF) and (**b**) S-0.5 specimens after eight cycles, and cross-sectional images of the crack and SAP voids in X–Z and X–Y planes.

Figure 12a,b show the three-dimensional image and the cross-sectional image of the crack and SAP voids in the X–Z and X–Y planes of the REF specimen and S-0.5 specimen, respectively, after eight cycles. The yellowish portions indicate autogenous healing products formed on the crack surface. As shown in the three-dimensional image and cross-sectional images in Figure 12, the healing products are uniformly distributed over the crack surface in the REF specimen, whereas they are concentrated around the SAP voids in the S-0.5 specimen. 

**Figure 13:**
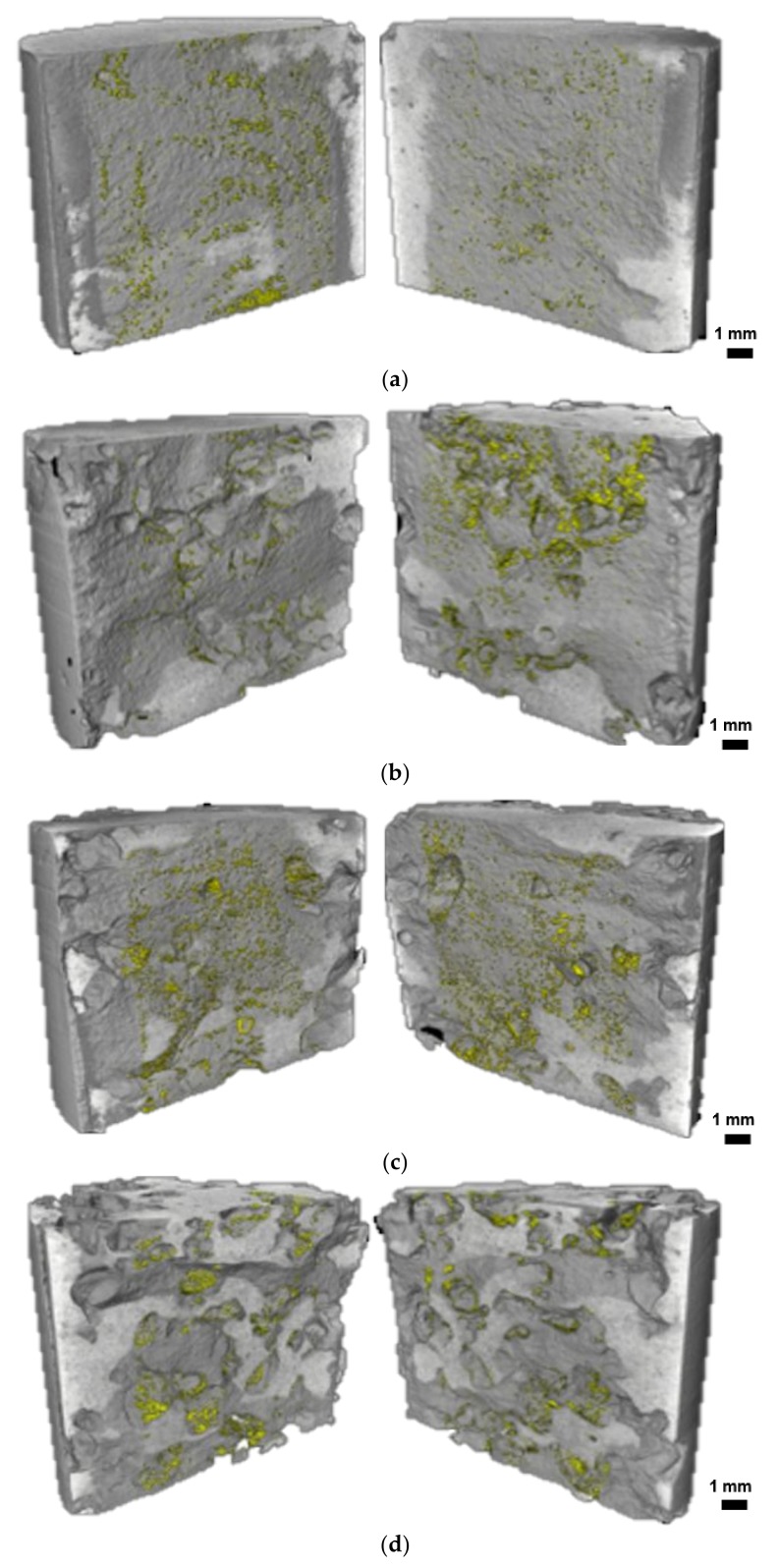
Three-dimensional images of split specimens; healing products are shown in yellow (**a**) REF; (**b**) S-0.5; (**c**) S-1.0; and (**d**) S-1.5 specimens.

Figure 13 shows the three-dimensional distributions of healing products generated on the crack surfaces and SAP voids of the specimens. It indicates that small volumes of healing products are produced at the crack surface of the REF and S-series specimens after eight wet/dry cycles. As shown in Figure 13a, the healing products in the REF specimen are distributed throughout the crack surface, whereas those in the S-series specimens are concentrated around the SAP voids, which is consistent with the two cross-sectional images of the S-0.5 specimen shown in Figure 12b. 

**Figure 14:**
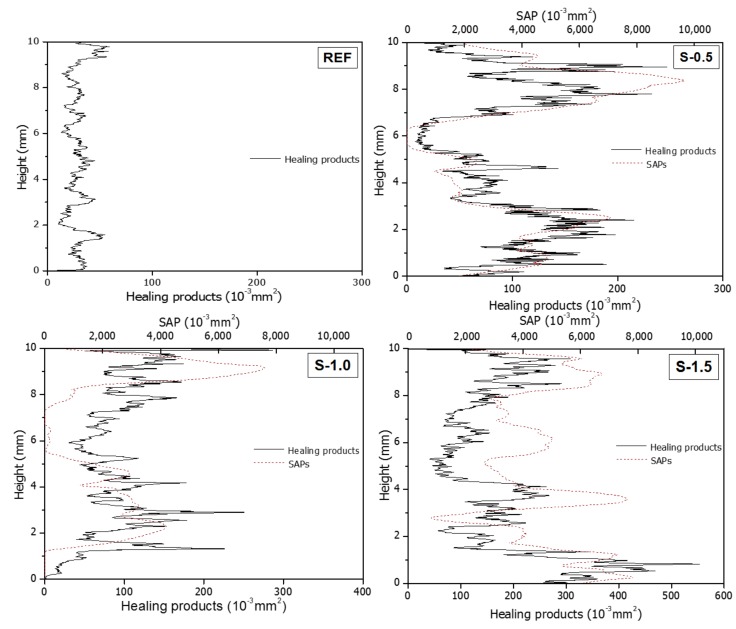
Areas of healing products and SAPs over the height of the specimens.

Figure 14 shows the distribution of the areas of the healing products and the SAPs directly connected to the crack as a function of the specimen height, obtained using the procedure that yielded Figure 11. The healing products in the REF specimen are similarly distributed regardless of the specimen height; their area is within the range 1.71 × 10^−3^ mm^2^ to 58.75 × 10^−3^ mm^2^. The specimens S-0.5 and S-1.0 include no SAPs at the heights 5.7–6.3 mm and 0.0–1.18 mm, respectively. The areas of the healing products in these sections are similar to that the REF specimen. In the sections where SAPs are located in the crack, however, healing products are concentrated around the SAP voids, and the graphs show increasing areas of healing products in the sections with increasing SAP void area. This is consistent with the result in the cross-sectional image in Figure 12, suggesting that the formation of healing products is promoted around the SAP voids. This is attributed to the good environments for the nucleation and growth of calcium hydroxide crystals at the SAP void surfaces, which releases calcium ions absorbed by the SAPs through the internal curing effect [42].

Table 3 shows the total volumes of the cracks before healing and of healing products, as well as the healing ratio calculated using Equation (3) after eight wet/dry cycles. The total pre-healing crack volume is determined by the crack ROI, defined as shown in Figure 10a, and the volume of the healing products is quantified by three-dimensional image analysis using the steps shown in Figure 11. Although the crack widths were controlled using silicone pads, as shown in Figure 2b, the crack volumes vary among the specimens. Because the crack volumes of the S-series specimens are determined by setting the crack ROI, which includes the crack and directly connected SAP voids, the crack volume is larger in the S-series specimens than in the REF specimen. The crack volume in S-1.0 is smaller than those in S-0.5 and S-1.5 because the silicone pad volume is larger in S-1.0 than in S-0.5 and S-1.5, and the absolute volume of healing products is also smaller in S-1.0 than in S-0.5. Table 3 indicates that the crack healing ratio is increased with increasing SAP dosage. This is because the internal curing effect and the corresponding autogenous healing performance are increased as the SAP dosage increases. 

**Figure 15:**
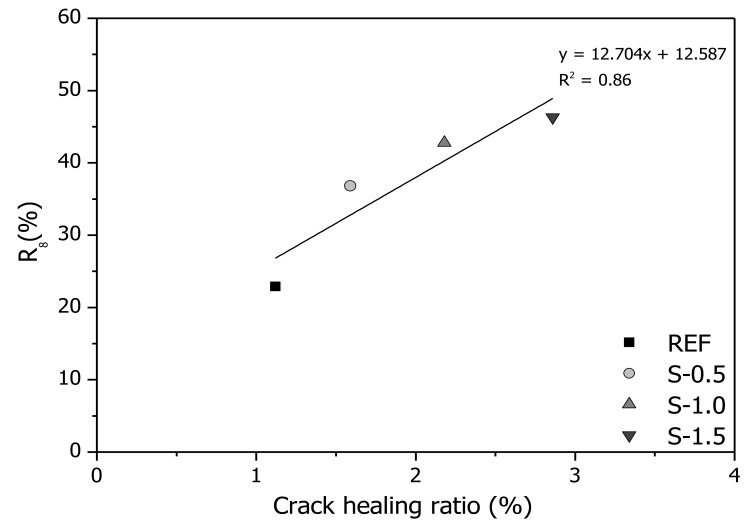
Relationship between reduction ratio of initial sorptivity at eighth cycle (*R*_8_) and crack healing ratio.

Figure 15 shows the correlation between the reduction ratio of initial sorptivity after eight cycles (*R*_8_) obtained from capillary water absorption testing and the crack healing ratio obtained from X-ray micro-CT analysis, along with the coefficient of determination from linear regression analysis. Both *R*_8_ and the crack healing ratio increase as the SAP dosage is increased. For example, *R*_8_ and the crack healing ratio of the S-1.5 specimen are 2.02 times and 2.55 times greater than those of the REF specimen, respectively. The linear regression analysis indicates that the reduction ratio of initial sorptivity is correlated well with the amount of healing products generated on the crack surface. This is because the initial sorptivity is decreased by the decreased absorption in the vertical and horizontal directions caused by healing product formation in the cracks, as confirmed by the X-ray micro-CT image analysis.

### 3.3. Compressive Strength

**Figure 16:**
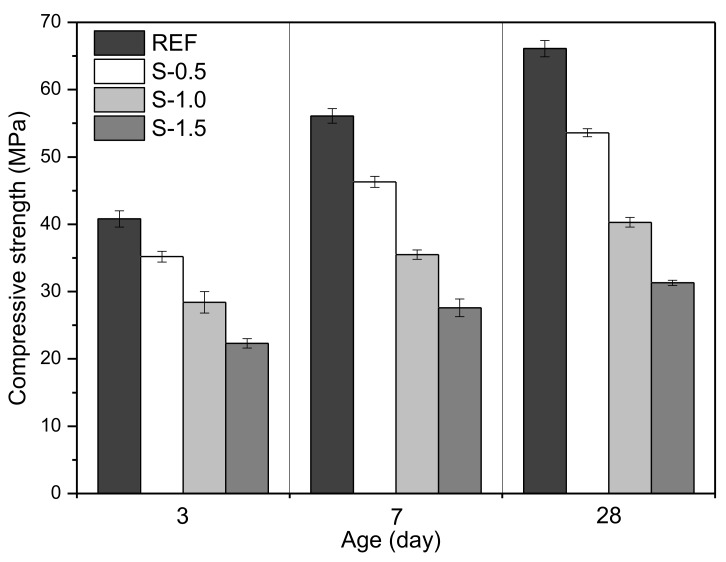
Compressive strength of specimens at 3, 7, and 28 days.

Incorporating SAPs leads to positive and negative effects, including both increased and decreased strength development of cementitious materials, due to internal curing effects, and the formation of more macro-voids by SAPs, respectively [19,36]. Figure 16 shows the mean compressive strengths and standard deviations of the REF, S-0.5, S-1.0, and S-1.5 specimens, at 3, 7 and 28 days. The 3-day compressive strengths of the REF, S-0.5, S-1.0, and S-1.5 specimens were measured as 40.8, 35.2, 28.4, and 22.3 MPa, respectively. The 7-day and 28-day compressive strengths of specimens were measured as 56.1 and 66.1 MPa for REF specimens, 46.3 and 53.6 MPa for S-0.5 specimens, 35.5 and 40.3 MPa for S-1.0 specimens, and 27.6 and 31.3 MPa for S-1.5 specimens. This shows that the compressive strength increased with decreased SAP dosage; this is because as SAP dosage increased and more macro-scale SAP voids formed, resulting in decreased compressive strength. This decrease was greater than the strength enhancement caused by the internal curing effect of SAPs. 

Considering the compressive strength results in this study, it is expected that the flexural strength of SAP-added specimens would be lower than that of SAP-free specimens, and that flexural strength would increase with decreased SAP dosage [19,33]. However, because the mechanical properties, including the strengths of SAP-added cementitious materials, are strongly dependent not only on the SAP properties, such as shape, particle size, and swelling ratio, but also on the mixture proportions of the cementitious materials, further study is needed to evaluate the mechanical properties of cementitious materials incorporating various types and dosages of SAPs. 

## 4. Conclusions

This study experimentally analyzed the effect of internal curing by SAPs on the autogenous healing performances of cementitious materials exposed to cyclic wet/dry conditions. Cement paste specimens with SAP dosages of 0%, 0.5%, 1.0%, and 1.5% by cement weight were prepared and single flexural cracks were induced. The autogenous healing performances were investigated via capillary water absorption testing. In the tests, 1 h wet conditions and 47 h dry conditions at the constant temperature of 25 °C and 50% RH were applied in eight cycles for 16 days. X-ray micro-CT scanning was performed to quantify the healing products and analyze their distribution. The findings of this study are summarized as follows:

1. The water absorption of all specimens was linearly proportional to the square root of measuring time within 1 h of capillary water absorption testing. The initial sorptivity, corresponding to the slope of the water absorption over the square root of time, tended to decrease with repeated wet/dry cycling.
2. The absorption rate in the second cycle *S*_2_ after the first cycle was decreased in all specimens because of the autogenous healing effect caused by further hydration of the un-hydrated cement particles at the crack surface. The reduction ratio of initial sorptivity of REF specimen after the second cycle (*R*_2_) was 19.8%, while those of the S-0.5, S-1.0, and S-1.5 specimens were 10.9%, 8.9%, and 10.7%, respectively. This is because less un-hydrated cement particles existed in the S-series specimens than in the REF specimen, because of the internal curing effect of the SAPs, thus yielding smaller decreases in *R*_2_ for the S-series specimens compared to that in the REF specimen.
3. As the SAP dosage increased, the reduction ratio of initial sorptivity was increased. *R*_8_ of the REF, S-0.5, S-1.0, and S-1.5 specimens were 22.9%, 36.8%, 42.8%, and 46.3%, respectively. This revealed that the SAPs located at the crack surface supplied the cracked cement matrix with additional moisture, which was used for further hydration in dry conditions, yielding the internal curing effect. Therefore, the autogenous healing performance was enhanced in the SAP-added specimens.
4. The initial sorptivity of all specimens decreased as autogenous healing progressed because the crack lengths were shortened by filling of narrow-width crack tips, which blocked the vertical moisture penetration path of water. In addition, the healing products generated on the crack surface and SAP void surface filled the capillary pores, thus blocking horizontal water penetration paths by the densification of the crack surface. These both contributed to decreases in initial sorptivity.
5. After eight wet/dry cycles, the REF specimen produced 0.29 mm^3^ of healing products with a crack healing ratio of 1.1%. The volumes of healing products and crack healing ratios of S-0.5, S-1.0, and S-1.5 specimens were 0.88 mm^3^ and 1.59%, 0.86 mm^3^ and 2.18%, and 1.67 mm^3^ and 2.86%, respectively. As the SAP dosage increased, the crack healing ratio increased and the initial sorptivity decreased. As the crack length was shortened by the formation of healing products at the crack tip, vertical penetration of moisture was reduced, thus decreasing the initial sorptivity.
6. X-ray micro-CT analysis revealed that the healing products were relatively uniformly distributed on the crack surface in the REF specimen, but they were concentrated around the SAP voids in the S-series specimens. Moreover, the distribution of the SAPs was similar to that of healing products as a function of the specimen height, suggesting that the internal curing effect of SAPs improved the autogenous healing performance of cementitious materials exposed to wet/dry cycling.
7. Both *R*_8_ and the crack healing ratio increased as the SAP dosage increased. The linear regression analysis indicated that the reduction ratio of the initial sorptivity was correlated well with the generation of healing products on the crack surface. This was because the healing products decreased the absorption in the vertical and horizontal directions, yielding reductions in the initial sorptivity.

This study focused on the autogenous healing of cementitious materials in hardening stages, a process mainly driven by the further hydration of unhydrated cement particles. Autogenous healing can be also manifested by the formation of healing products over the longer period, and in fully hardened materials, which are areas where further study is needed.

## Figures and Tables

**Table 1:** Chemical composition and LOI of Portland cement used in the test (mass%).

SiO_2_	Al_2_O_3_	Fe_2_O_3_	CaO	MgO	SO_3_	K_2_O	Na_2_O	LOI ^1^
20.5	4.97	3.02	61.8	2.71	2.35	0.72	0.33	2.36

^1^ LOI: loss on ignition.

**Table 2:** Mixture proportion of cement paste specimens used in the test.

Specimen	Cement (kg/m^3^)	d% SAP ^1^ (%)	SAP (kg/m^3^)	Water (kg/m^3^)	Water in SAP (kg/m^3^)
REF	1498.2	0.0	0.0	524.4	0.0
S-0.5	1498.2	0.5	7.5	524.4	75.0
S-1.0	1498.2	1.0	15.0	524.4	150.0
S-1.5	1498.2	1.5	22.5	524.4	225.0

^1^ SAP dosage by weight of cement.

**Table 3:** Crack healing ratios of specimens.

Specimen	Total Volume of the Crack (mm^3^)	Total Volume of the Healing Products (mm^3^)	Crack Healing Ratio (%)
REF	25.53	0.29	1.1
S-0.5	55.80	0.88	1.6
S-1.0	39.50	0.86	2.2
S-1.5	58.32	1.67	2.9
